# Fatal association of COVID‐19 and acute type A aortic dissection

**DOI:** 10.1002/ccr3.5617

**Published:** 2022-03-22

**Authors:** Rana Irilouzadian, Hossein Salehi Omran, Toktam Alirezaei

**Affiliations:** ^1^ Faculty of Medicine Shahid Beheshti University of Medical Sciences Tehran Iran; ^2^ Cardiology Department of Shohadaye‐Tajrish Hospital Shahid Beheshti University of Medical Sciences Tehran Iran

**Keywords:** aortic dissection, cardiovascular disorders, coronavirus, COVID‐19

## Abstract

Type A aortic dissection is a catastrophic event that requires prompt diagnosis and intervention to save the patient. It seems that type A aortic dissection in COVID‐19 patients has increased severity, and even with immediate diagnosis, it has a high mortality.

## Graphical abstract

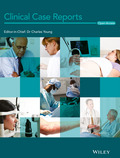

## 1 INTRODUCTION

Corona virus disease 2019 (COVID‐19) is a respiratory disease caused by severe acute respiratory syndrome coronavirus 2 (SARS‐CoV‐2). More than 250 million people have been infected around the world, and unfortunately, more than five millions of them lost their lives to date (November 2021).^1^ COVID‐19 can present in a wide range of clinical manifestations, more commonly as respiratory symptoms such as shortness of breath and cough. Furthermore, it can manifest as cardiovascular symptoms such as palpitations and chest pain^2^ and cardiovascular complications such as aortic dissection.^3^ Aortic dissection is described as a tear in the intima layer of aortic wall which allows blood to flow and accumulate between aortic layers and can propagate along the length of aorta. It can manifest as various symptoms such as chest pain, weakness, fatigue and unequal extremity pulses, and blood pressures.^4^ Despite all advances in diagnostic and treatment fields of medicine, aortic dissection has still a high rate of mortality which makes it extremely considerable.^5^ In this report, we aimed to present our patient with aortic dissection and recent COVID‐19 and report her final outcome.

## 2 CASE HISTORY/EXAMINATION

A 46‐year‐old woman was admitted to our emergency room with acute‐onset chest pain which was retrosternal and radiating to the left arm and between two shoulders and chest heaviness. The pain was continuous from 2 hours before admission. In addition, she mentioned nausea, vomiting, and cold sweat. The patient was nonsmoker and had a past medical history of stage 1 hypertension based on ACC/AHA 2021 hypertension guidelines for about 3 years and hyperlipidemia. She was under a single‐drug treatment with a daily dosage of 25 mg of losartan tablet for hypertension and was under control. She was also affected by COVID‐19 3 weeks ago and underwent outpatient treatment with remdesivir for 5 days during which the patient did not experience fluctuations in her blood pressure.

On physical examination, a systolic blood pressure difference of 40 mmHg between arms (140/80 mmHg in right arm versus 100/80 mmHg in left arm) was detected by sphygmomanometer in the assessment of vital signs. Her heart rate was 82 beats per minute. Respiratory rate was 16 breaths per minute, and arterial O2 saturation was 96%. Pulmonary and heart auscultation were unremarkable. A discrepant pulse between left and right radial artery was felt, where the left radial pulse was weaker. Routine neurological examination was normal, and the power in all of the limbs was 5/5, with a Glasgow coma scale of 15/15. Pupils were 3 mm, bilaterally equal and reactive to light, the abdomen was soft and lax with no tenderness.

### 2.1 Differential diagnosis, investigations and treatment

**FIGURE 1:**
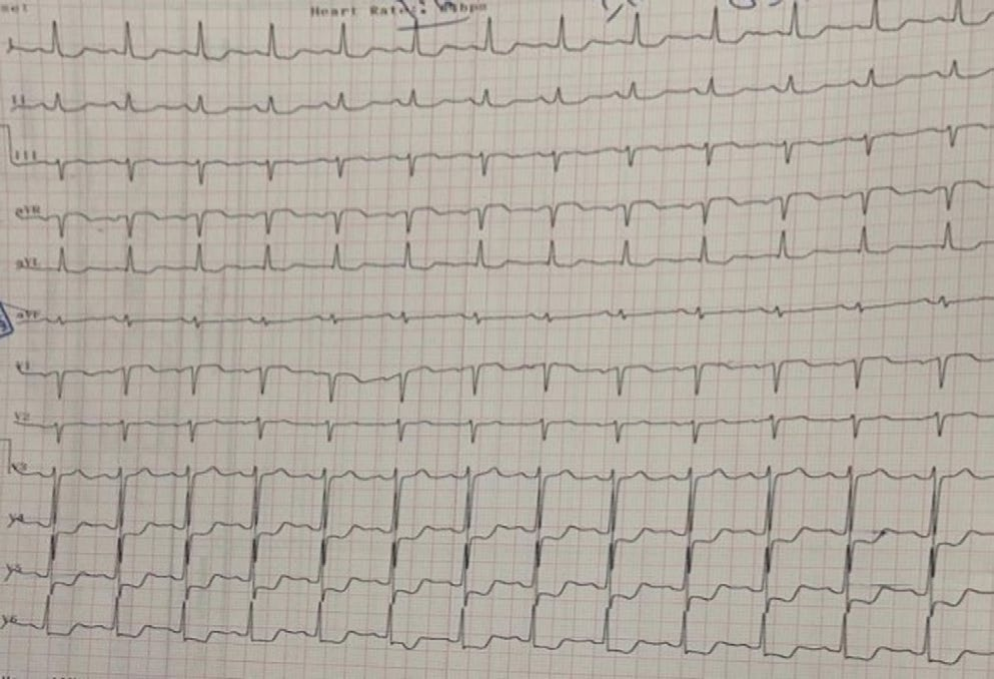
Initial ECG of patient: normal sinus rhythm, normal axis, ST elevation in aVR and V1 leads and ST depression in I, aVL, V4‐6 leads

Initial ECG showed normal sinus rhythm, normal axis with ST elevation in aVR and V1 leads and ST depression in I, aVL, V4‐6 leads (Figure 1). According to the patient's presentation with chest pain, unequal radial pulses, a difference in blood pressures of arms and her ECG, aortic dissection was the most likely diagnosis, and an emergent aortic computed tomography (CT) angiography was requested for the patient.

In laboratory tests, D‐dimer was 4820 ng/ml (reference up to 600), serum ferritin 417.4 ng/ml (reference 10–291), lactate dehydrogenase 666 U/L (reference 230–480), troponin 0.2 ng/ml (normal population <0.05), CPK 58 U/L (24–170), CK‐MB 20 U/L (reference 1–24), C‐reactive protein 16 mg/L (reference up to 5.9), and ESR 26 mm/h (reference up to 20 in below 50 years old patients). Other para‐clinical tests were normal.

**FIGURE 2:**
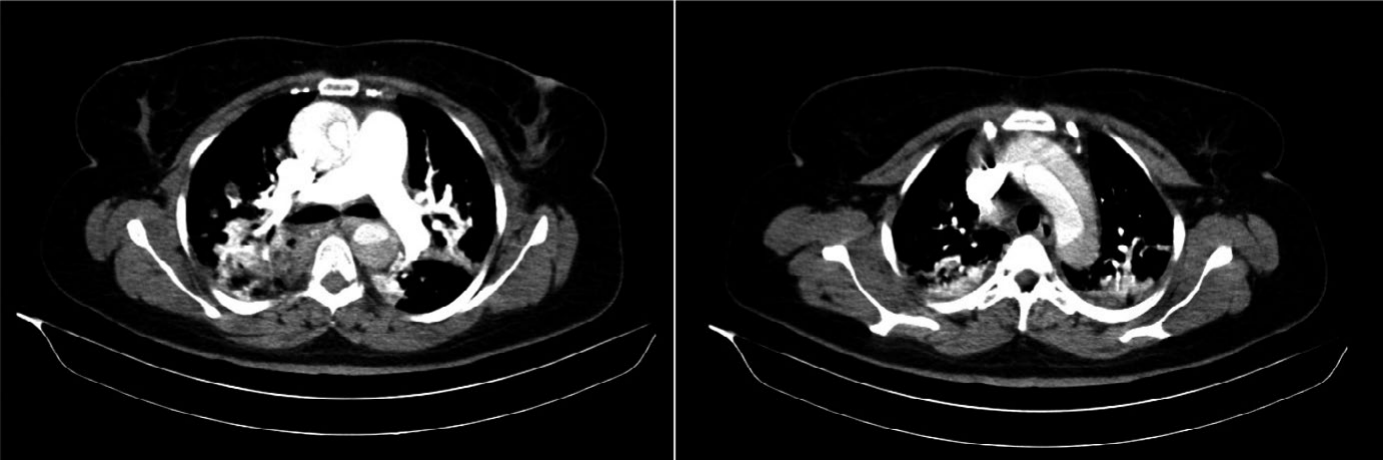
Aortic CT angiography: type A according to Stanford classification and type 1 according to DeBakey classification

Following aortic CT angiography, aortic dissection type A according to Stanford classification and type 1 according to DeBakey classification was diagnosed for the patient. The intimal flap that started at aortic valve, extending to ascending aorta, aortic arch, descending thoracic aorta, abdominal aorta to right common iliac artery and then terminating in right external iliac artery, was noted. Also, dissection flap extension to right carotid artery, right common carotid artery and left subclavian artery was seen (Figure 2). Right coronary artery was supplied by true lumen and left main artery was supplied by false lumen. The chest CT scan showed patchy ground glass opacity in anterior segment of superior and middle lobes of right lung consistent with COVID‐19 infection.

Due to diagnosis of aortic dissection, the patient was a candidate for emergency surgery and due to inadequate facilities in our hospital, she was prepared to be transferred to another hospital, but as soon as she entered the ambulance, she became bradycardic that led to asystole. Cardiopulmonary resuscitation (CPR) was performed and she was returned to our hospital. The patient underwent intubation and atropine injection. Then, the patient was successfully recovered after a few minutes and she regained consciousness. After an hour, she was transferred to another hospital for emergency surgery.

### 2.2 Outcome and follow‐up

The patient was unfortunately expired before entering the operating room.

## 3 DISCUSSION

In this report, we described a 46‐year‐old woman with past medical history of stage 1 hypertension which was under control, hyperlipidemia and recent COVID‐19 whom presented with acute chest pain and cold sweat. The patient reported no traumatic events and no history of connective tissue disorders. Her clinical examination showed unequal radial pulses and a blood pressure difference between arms. During patient's hospitalization, her blood pressure was within the normal range. The laboratory tests showed elevated D‐dimer, troponin, ESR, and CRP. The diagnosis and management of aortic dissection in early stages is of importance since it has numeric manifestations and can mimic other life‐threatening events such as myocardial infarction and pulmonary embolism.^6^ In this regard, patient death can result from delayed treatment and/or increased diagnostic interval, mostly the latter. Our patient had a systolic blood pressure difference between two arms and her initial ECG was compatible with left main coronary artery involvement. So, our first suspicion was type A aortic dissection. Therefore, we performed aortic CT angiography without hesitation and the diagnosis of type A aortic dissection was established promptly. As we mentioned before, our hospital did not have any cardiothoracic surgeon and the patient transfer to another hospital was arranged but unfortunately, she became bradycardic and then asystolic. After performing CPR and stabilizing the patient, she was transferred to another hospital for emergency surgery but she died before entering the operating room.

It has been suggested that complications and mortality of cardiovascular emergencies and number of severe cases had been increased during the COVID‐19 pandemic.^7^, ^8^ Moreover, in another study, postoperative mortality was higher in COVID‐19 patients who needed cardiovascular surgery, compared to the patients without COVID‐19.^9^ Type A aortic dissection in COVID‐19 patients seems to associate with increased severity that even with immediate and proper diagnosis, it has higher mortality.

Silvestri et al.,^10^ reviewed seventeen cases of aortic pathology in patients with clinically suspected or PCR‐confirmed COVID‐19 and also reported hypertension as the most frequent comorbidity; they suggested a potential link between COVID‐19 and aortic dissection. There are some potential mechanisms for arterial pathology in COVID‐19 patients. SARS‐CoV‐2 has spike proteins on its surface that binds a receptor which is expressed in the endothelium called angiotensin converting enzyme 2 (ACE‐2). This means that SARS‐CoV‐2 can injure vascular endothelium in the body.^11^ SARS‐CoV‐2 downregulates ACE‐2 which leads to overactivation of classical renin‐angiotensin system (RAS) and vasoconstriction.^12^ ACE Inhibitor (ACEI) and angiotensin receptor blocking (ARB) drugs, which are used commonly for hypertension as in our patient, upregulate ACE‐2 expression that can potentially increase the vascular entry of and injury by SARS‐CoV‐2. On the contrary, upregulation of ACE‐2 can have vasodilatory and anti‐inflammatory effects as a result of conversion of angiotensin II to angiotensin 1–7.^13^ However, in a study of 1128 hospitalized patients with COVID‐19, those who took ACEI/ARB drugs had a lower all‐cause mortality than those who did not take.^14^

Another possible cause of arterial dissection in COVID‐19 patients can be cytokine storm and inflammatory responses which leads to endothelial dysfunction.^15^ Inflammation may cause rupture of atherosclerotic plaque which can lead to dissection.^16^ Studies have shown that the number of patients with aortic dissection were increased during the influenza season.^17^, ^18^ Akgul et al.^19^ presented an aortic dissection in a COVID‐19 patient which during the aortotomy, they noticed significant aorta wall thickening as seen in inflammatory aortic pathologies. Their finding is consistent with the potential association of inflammation caused by SARS‐CoV‐2 with aortic dissection. As it has been suggested before, SARS‐CoV‐2 is a virus that causes multi‐organ diseases and can manifest as life‐threatening events.^12^ Therefore, it is important to evaluate the association between COVID‐19 and aortic dissection and the pathophysiology of it. Further studies are needed to establish this association.

## CONFLICT OF INTEREST

The authors have no conflict of interest to declare.

## AUTHOR CONTRIBUTIONS

Rana Irilouzadian involved in conceptualization, writing original draft, review, and editing. Hossein Salehi Omran involved in data collection, writing original draft. Toktam Alirezaei involved in management of the patient, supervision, review, and editing.

## ETHICAL APPROVAL

As the patient was deceased, a written informed consent was obtained from the patient's next of kin. All of the authors declare that confidentiality of the patient was respected.

## CONSENT

As the patient was deceased, a written informed consent was obtained from the patient's next of kin to publish this report in accordance with the journal's patient consent policy and all of the authors declare that confidentiality of the patient was respected.

## Data Availability

The data that support the findings of this study are available on request from the corresponding author.
